# Carbonisation temperature dependence of electrochemical activity of nitrogen-doped carbon fibres from electrospinning as air-cathodes for aqueous-alkaline metal–air batteries†

**DOI:** 10.1039/c9ra03805a

**Published:** 2019-08-30

**Authors:** Markus Gehring, Hermann Tempel, Alexandre Merlen, Roland Schierholz, Rüdiger-A. Eichel, Hans Kungl

**Affiliations:** Forschungszentrum Jülich GmbH, Institute of Energy and Climate Research – Fundamental Electrochemistry (IEK-9) 52425 Jülich Germany m.gehring@fz-juelich.de +49 2461 61-4155 +49 2461 61-96689; Rheinisch-Westfälische Technische Hochschule Aachen, Institute of Physical Chemistry 52056 Aachen Germany; IM2NP, CNRS, Aix-Marseille Université, Université de Toulon Toulon France

## Abstract

Poly-acrylonitrile (PAN)-derived carbon fibres were characterised as air electrode frameworks for aqueous-alkaline metal–air batteries, focussing on the influence of the carbonisation temperature on the structure and electrochemical properties. Elemental composition, (atomic) structure, electrical conductivity, and electrochemical performance related to the oxygen reduction were investigated for electrodes carbonised in the range from 300 °C to 1400 °C. Chemical and structural properties were analysed using elemental analysis, XPS, SEM, and Raman spectroscopy; electrical conductivities of the fibre networks were examined by four-point probe measurements. Electrochemical properties were evaluated using linear sweep voltammetry in 6 M KOH by the open circuit potentials, the cathodic current densities at given overpotentials, and required overpotentials at given current densities. The highest current density was obtained from fibres carbonised at 850 °C. The connection between the fibre characteristics and electrochemical properties are discussed, highlighting the importance of the nitrogen bonding state. The results provide a base for thedevelopment of high performance air electrodes.

## Abstract 2

Nitrogen-doped carbon fibres derived from electrospun poly-acrylonitrile display an optimum catalytic activity towards the oxygen reduction reaction in aqueous-alkaline metal–air batteries, when carbonised under argon at 850 °C.

## Graphical abstract

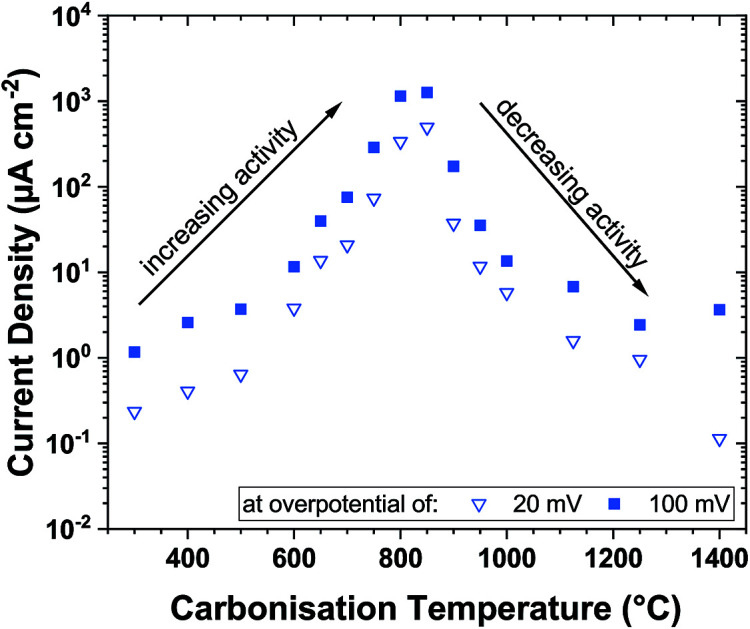

## 1 Introduction

Over the last two decades, metal–air batteries have (re-)emerged as a potential energy storage system, because of their high theoretical energy densities; environmental friendliness; and abundant, low-cost materials. Primary Zn–air systems have been commercialised for low-power applications, but recent developments in the energy sector have led to a demand in better performing and rechargeable battery systems.^1–3^

Recent reports on primary and secondary aqueous-alkaline metal–air battery systems, such as the Zn–air,^1^ Fe–air,^4,5^ and Si–air^5^ systems, have stressed the points that currently available anodes are performing well and that the full cell performance is limited mainly by the air cathode.^6^ During discharge the overall cell voltage is reduced as a result of the high over-potentials in the cathode, and there is a demand for optimisation of the employed catalyst materials as well as the electrode architecture.^1,2^

Typically, air cathodes consist of several layers: a hydrophobic membrane, such as porous PTFE; a Ni-based current collector; a gas diffusion layer, composed of pressed carbon powder and PTFE; and a catalytically active layer.^1,7^ Improvements and further development of the air cathode can follow either of two routes: an iterative improvement of the state-of-the-art design or fundamentally new design approaches. In this work electrodes composed of a mat of mechanically self-supporting electrospun carbon fibres from poly-acrylonitrile (PAN) are presented as such a new-design approach.

Electrospinning, unlike traditional wet-spinning, results in non-woven fibre mats composed of fibres with diameters in the sub-micrometre range and a large surface areas.^8^ As a result, the carbon fibre mats derived from electrospinning feature a number of advantages over the—traditionally pressed—carbon powders: the pore structure and the surface properties can be tailored to the application at hand.^9^ The resulting carbonised fibre mats are self-standing and extremely light-weight. Most importantly, chemical composition, structure, and surface morphology of the fibres can be predetermined by choosing appropriate precursors and processing conditions.^9^

The precursors are, typically, polymer solutions that can even be further enhanced with additives, such as catalysts, that can be directly integrated into the fibres.^9–11^ This may reduce the amount of additional preparation steps. The precursor material can be chosen from a wide range of polymers, thus the final composition of the fibres can be influenced *a priori*. Most importantly, hetero-atom doping becomes easily accessible by using polymers that contain *e.g.* nitrogen, such as PAN.^9,12,13^

PAN is a thoroughly investigated model material both for the production of carbon fibres as well as electrospinning.^8^ The carbon fibres are obtained by heat treatment of the polymer fibres under protective atmosphere. The complex carbonisation mechanism of PAN has been studied extensively by many groups since initial reports in the early 1960s by Shindo.^14,15^

In terms of electrochemistry, PAN is a suitable precursor material for carbon, as the carbon yields are high; the surface area of the fibres increase with carbonisation temperature;^16^ and, most importantly, PAN derived carbon typically retains at least some of its nitrogen, depending on the carbonisation temperature applied.^17^ Nitrogen-doped carbon has an inherent auto-catalytic activity towards oxygen redox chemistry. In the last decades, nitrogen-doped carbon has been proposed as a possible metal-free catalyst for the oxygen reduction reaction (ORR) and the oxygen evolution reaction (OER). Accordingly, doping with other hetero-atoms has been shown to enhance the activity, as well. This includes boron-doping,^18–20^ sulphur-doping,^20^ and phosphorous-doping.^20,21^ All of these dopants have been shown to influence the catalytic activity, but also structural properties, such as the active surface area.^8^ The mechanisms behind the activity, however, have remained a point of discussion.^22–27^

The catalytic route for the ORR on platinum is well understood.^28^ However, it has been established that the catalytic route on nitrogen-doped carbons is a different one,^29^ and the activity, in very general terms, originates from the interaction of the carbon π-system with the nitrogen lone pair.^30^ The nitrogen doping increases the electron density near the carbon's Fermi level, which facilitates the breaking of the O–O bond by transferring electrons from the carbon π-system to the oxygen's anti-bonding 2σ_p_^*^
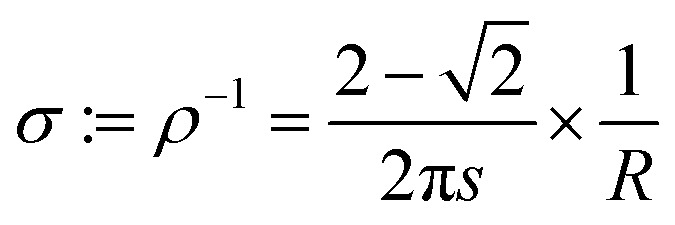
 or 2 π^*^ orbitals.^30,31^ The overall ORR equation in aqueous-alkaline media is given by eqn (1).

1 O_2(g)_ + 2H_2_O_(l)_ + 4e^−^ ⇌ 4OH^−^_(aq)_; *E*_0_ = 0.04 V *vs.* RHE^3,29^

Initially, oxygen will adsorb onto a β-carbon site (eqn (2))

2 O_2(aq)_ ⇌ O_2(ads)_

There are different modes in which oxygen can adsorb to a (catalyst) surface. More recent calculations have found that the end-on oxygen adsorption (Pauling model) is energetically favourable to side-on adsorption (Yeager model) on nitrogen-doped carbon.^29^ The subsequent reaction is determined primarily by the adsorption mode, with end-on adsorption leading to a so-called associative mechanism and side-on adsorption to an dissociative mechanism.

In the associative mechanism, as a first step, the adsorbed oxygen forms adsorbed hydroperoxide (eqn (3)) and the O–O bond is broken subsequently, as detailed by eqn (4). This mechanism corresponds to the energetically favourable 4e^−^ mechanism.^32^ The peroxide then disproportionates (eqn (4)), forming adsorbed hydroxide (eqn (5)). Lastly, the remaining hydroxide desorbs (eqn (6)).

3 O_2(ads)_ + H_2_O_(l)_ + e^−^ ⇌ OOH_(ads)_ + OH^−^_(aq)_

4 OOH_(ads)_ + e^−^ ⇌ O_(ads)_ + OH^−^_(aq)_

5 O_(ads)_+H_2_O_(l)_ + e^−^ ⇌ OH_(ads)_ + OH^−^_(aq)_

6 OH_(ads)_ + e^−^ ⇌ OH^−^_(aq)_

However, both experimental and theoretical studies have not reached a conclusion, as to which nitrogen species is causing the catalytic activity. Both types of studies have found arguments for attributing the enhancement to either pyridinic nitrogen^33^ or graphitic nitrogen.^23^ Based on DFT calculations, Kim and co-workers have proposed a ring-opening mechanism, involving initially graphitic nitrogen, which becomes pyridinic nitrogen upon ring-opening, in an attempt to reconcile the two points of view.^32^ Also, these results underline the importance of edge-type nitrogen, which has generally been found to be more active towards ORR.^30^

In the dissociative mechanism the oxygen O–O bond is broken as the first step (eqn (7)) followed by the 2e^−^ mechanism (per oxygen atom) (eqn (5) and (6)).^29,32^

7 O_2(ads)_ ⇌ 2O_(ads)_

Overall, using PAN-derived electrospun carbon fibres as an air-cathode framework is a sensible approach, as has been demonstrated by other groups,^13^ but a deeper understanding of the unmodified material is still required, when high performance is sought. This entails a detailed evaluation of the parameters of both the electrospinning process and the post-processing.

In this paper the relationship between the nitrogen content; bonding situation; fibre morphology and structure; and the electrochemical properties are investigated. The main focus are the gradual changes in the PAN-derived fibres during carbonisation in a wide carbonisation temperature range from 300 °C to 1400 °C.

## 2 Experimental

### 2.1 Synthesis of carbon fibres and electrode preparation

Solutions of 10 wt% poly-acrylonitrile (PAN, *M*_W_ = 150 000 g mol^−1^; BOC Sciences, USA) in *N*,*N*-dimethylformamide (DMF; Merck, France) were prepared by stirring the mixture at room-temperature for a minimum of three days. The solution was filled into a syringe and pumped into the electrospinning chamber (FEM Technologies, The Netherlands) with a fixed flowrate of 40 μL min^−1^. The distance between the nozzle and the collector was set to 15 cm. The voltage at the nozzle was set to 21 kV and at the collector to −4 kV. The rotating drum collector was covered with non-adhesive paper and its rotation speed was set to 1500 rpm.

For all samples, the temperature in the spinning chamber was 25 °C with a relative humidity of 40%. The spinning time was 6 h per sample. The obtained fibre mats were stabilised in air at 250 °C overnight and subsequently carbonised at temperatures between 300 °C and 1400 °C for 3 h under argon. The heating rate was 5 K min^−1^ and the cooling rate was 3.33 K min^−1^.

The carbonised samples were cut into pieces of approximately 3 cm by 3 cm. A hydrophobic PTFE membrane (PMV10L; Porex, USA) was applied by means of hot rolling at 120 °C.

### 2.2 Physical characterisation

SEM images were recorded using a Helios NanoLab 460F1 (FEI Europe) with an acceleration voltage of 20 kV. The samples were attached to the sample holder using a double-faced graphite tape and conductivity was further improved by applying a copper tape to both the sample and the graphite tape. The fibre diameters were determined from the resulting images using a total of 100 fibres per sample from five representative spots of each sample.

Raman spectra were obtained using a Bruker Senterra at room-temperature in ambient atmosphere. The excitation wavelength was 532 nm with a power of 2 mW. The signals of two subsequent measurements of 30 s each were added together, to improve the signal to noise ratio (Co-addition mode).

For the elemental analysis 2 mg of sample was burned in the elemental analyser (VarioelCube, Elementar) for both CHN and O analyses. The signal was detected using a heat conductivity detector.

X-ray photo spectroscopy (XPS) was performed with a Phi5000 VersaProbe II (ULVAC-Phi Inc., USA). Spectra were recorded using Al-Kα radiation (1.486 keV) at 50 W with a resolution of 0.1 eV and a spot-size of 200 μm.

Electrical resistance was recorded using a custom-made four-point probe device with a square arrangement of stainless steel tips with a tip distance (*s*) of 2.54 mm. Resistance values were detected using a table top multimeter (2701 Integra, Keithley, USA). The conductivity *σ* (S cm^−1^) was calculated from the measured resistance *R* (Ω sq^−1^) using the equation used for the 3D bulk-resistivity *ρ* (Ω cm) in the square set-up (eqn (8)):^34^8
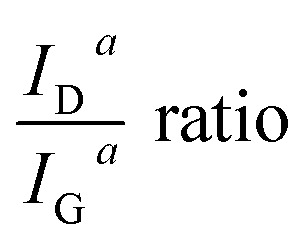


### 2.3 Electrochemical investigation

Aqueous 6 M KOH was used as electrolyte. Appropriate amounts of KOH (85%; VWR, France) were dissolved in de-ionised water (0.055 μS cm^−1^; Purelab flex, Elga Veolia, United Kingdom).

The electrochemical investigation was performed in a half-cell setting, using a commercially available cell (FlexCell-PP, Gaskatel, Germany). The counter electrode was a platinum wire and a mercury/mercury oxide electrode (MMO; ALS, Japan) was used as a reference electrode. Unless stated otherwise, the cell was assembled and equilibrated at OCP for 1 h. Linear potential sweep voltammetry (LSV) was performed with a scan rate of 1 mV s^−1^ in a potential window between 0.2 V *vs.* MMO and −0.5 V *vs.* MMO. Measurements were performed sequentially after 1 h, 2 h, 4 h, 8 h and 12 h for each sample, between each measurement cells were left at OCP.

## 3 Results and discussion

### 3.1 SEM analysis of fibres

**Fig. 1:**
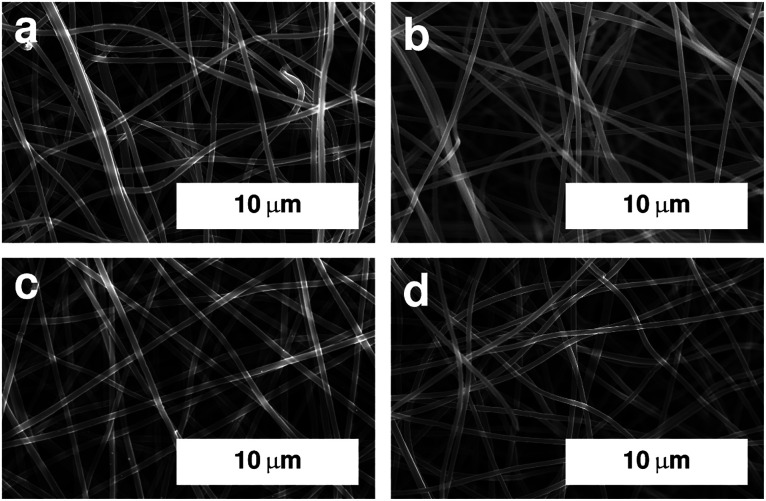
SEM images of electrospun PAN fibres after stabilisation in air at 250 °C and carbonisation under argon at (a) 600 °C, (b) 800 °C, (c) 1000 °C, and (d) 1250 °C. Image for sample carbonised at 400 °C is shown in Fig. S1.†

The overall morphology of the fibre mats after carbonisation was investigated using SEM. Images of selected samples are shown in Fig. 1. All samples exhibit a fibrous morphology with smooth surfaces. The fibre mats underwent significant macroscopic shrinking during the carbonisation process, which, microscopically, resulted in a lower volume of in-between space in the fibre mats.

**Fig. 2:**
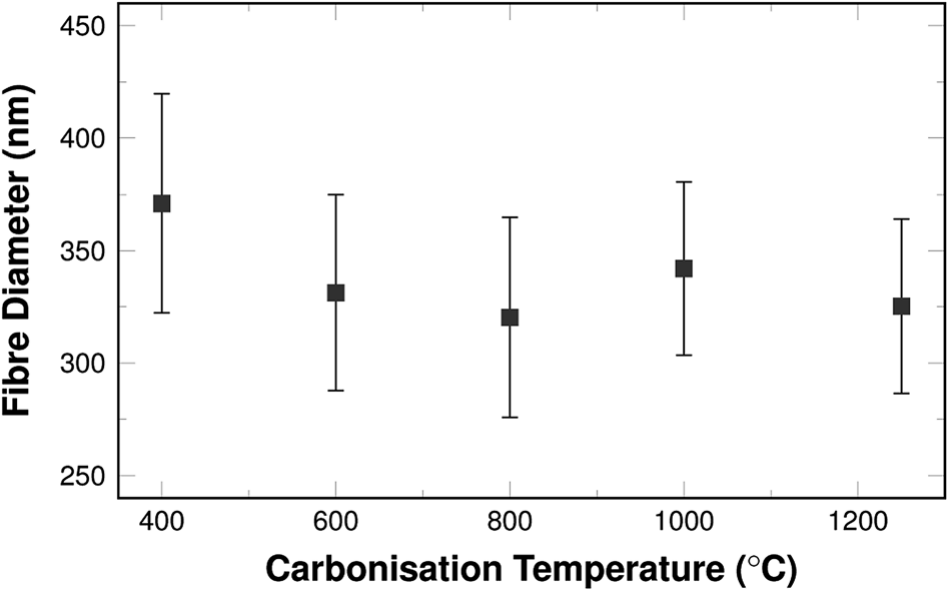
Fibre diameters as a function of carbonisation temperature.

The shrinking of the fibres is also reflected in the fibre diameters themselves. The mean diameters decrease with carbonisation temperature as shown in Fig. 2.

The diameters vary between 270 nm and 470 nm. This is well within the typical range that can be found in literature for comparable fibre systems.^35^ The shrinking is most pronounced in the temperature range of 400 °C and 600 °C and the diameters are similar for samples carbonised above 600 °C.

### 3.2 Elemental composition and X-ray photoelectron spectroscopy (XPS)

Elemental analysis shows that the relative carbon content in oxidatively stabilised fibres ((52.7 ± 0.1) wt%) is lower than in PAN powder ((66.6 ± 0.2) wt%) and PAN fibres (66.1 ± 1.3) wt%). This is directly related to the oxidation of the fibres. The elemental composition of the as spun fibres is equivalent to that of the powder, which suggests that the chemical composition does not change during the electrospinning process.

**Fig. 3:**
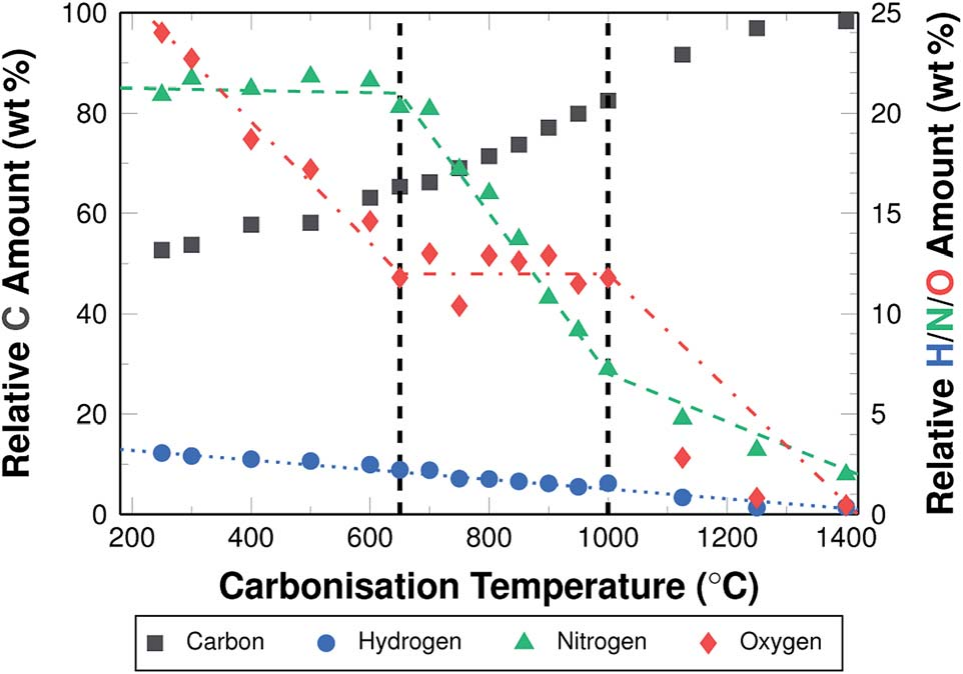
Elemental analysis (H, C, N, and O) of stabilised fibre mats and carbonised fibre mats.

During carbonisation the relative carbon content increases. The increase is higher for higher carbonisation temperatures. The carbon content is 53.7 wt% for fibres carbonised at 300 °C, and 98.4 wt% for fibres carbonised at 1400 °C. Fig. 3 shows the different elemental compositions for all investigated temperatures.

It becomes clear that the increase of the relative carbon content is the result of a loss of nitrogen, oxygen, and hydrogen. The changes in oxygen and nitrogen contents differ in slope depending on the carbonisation temperature. Three distinctive sections can be identified and assigned to three temperature ranges. Between 300 °C and 650 °C the oxygen content is reduced from 24 wt% to 14 wt%, while the nitrogen content remains stable at about 21 wt% . In the second section between 650 °C and 1000 °C, the oxygen content is further reduced only slightly, to 11 wt%, while the nitrogen content decreases from 21 wt% to about 7.5 wt%. At temperatures above 1000 °C, the oxygen content is reduced significantly to below 1 wt%, and to almost 0 wt% when carbonising at 1400 °C. The nitrogen content is further reduced, at a lower rate than before, to 2.5 wt%, which remain in the material. Hydrogen is removed slowly but steadily in the entire temperature range from 3 wt% to almost 0 wt% (Fig. 3).

It has to be noted, that not only the relative amount of hetero-atoms is reduced, but also the absolute weight of the fibres. This weight loss results from the removal of oxygen, nitrogen, and hydrogen as gases in various combinations, but foremost as N_2_, H_2_, NH_3_, HCN, and H_2_O.^15,36^

**Fig. 4:**
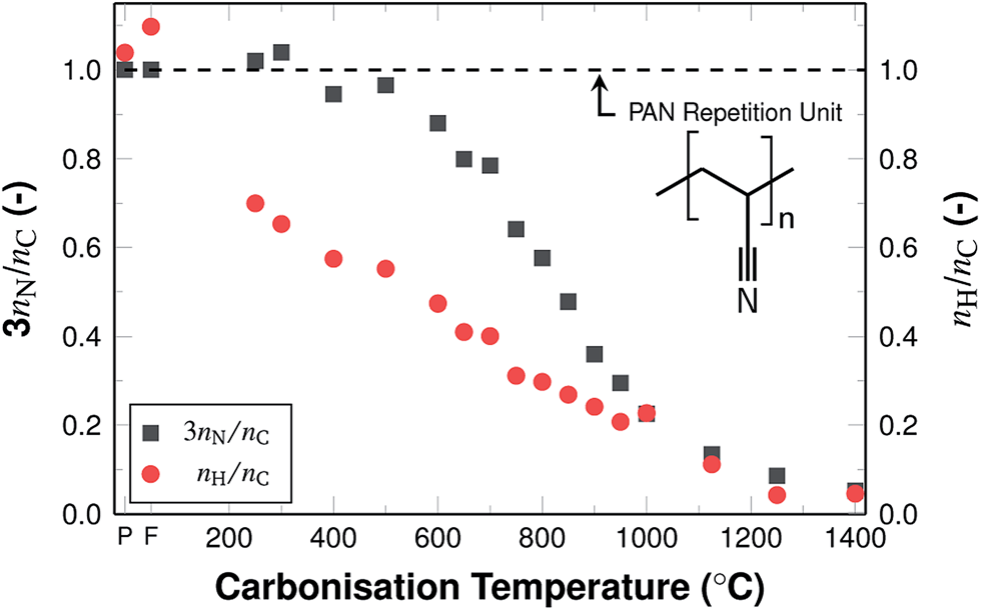
Ratios of amounts of substance of nitrogen and carbon (grey) and hydrogen and carbon (red), relative to the ratio in the PAN repetition unit (black dashed line). P and F indicate PAN powder and PAN fibres, respectively.

The ratios of amounts of substance from the elemental analysis (Fig. 4), show changes in the chemical composition of the material compared to the PAN repetition unit. This implies pronounced changes in the atomic structure of the fibres from polymeric PAN towards graphite-like carbon. This transformation is indicated by the increasing deviation of the initial 3*n*_N_/*n*_C_ ratio, where *n*_N_ is the amount of substance of nitrogen and *n*_C_ that of carbon. The ratio increases slightly when comparing as-spun fibres to stabilised fibres, as a result of the oxidation, but decreases from 1.03 to 0.05 throughout the investigated temperature range during carbonisation.

The 3*n*_N_/*n*_C_ ratio follows a sigmoidal trend during carbonisation. It can be described by three phases, based on the carbonisation temperature, similar to the overall development of the nitrogen content (Fig. 3). The decrease progresses rather slowly at temperatures below 600 °C. At higher temperatures the decrease is more pronounced and the values decrease from 0.9 at 600 °C to 0.2 at 1000 °C. At temperatures above 1000 °C the ratio is reduced to very low values at 1400 °C, as the nitrogen has been almost completely removed.

The ratio of hydrogen to carbon (*n*_H_/*n*_C_) is higher than that of the repetition unit only for un-stabilised PAN repetition units. The higher values may be the result of various influences such as PAN end groups, adsorbed species (such as water), measurement errors, or impurities. The ratio for stabilised fibres is 0.7 as a result of the oxidative stabilisation, where the nitrile groups polymerise to conjugated imines and the related removal of gaseous H_2_ and NH_3_ takes place. The ratio values decrease from 0.7 to 0 almost linearly. This removal process results in an aromatic ring structure.^15,37^

**Fig. 5:**
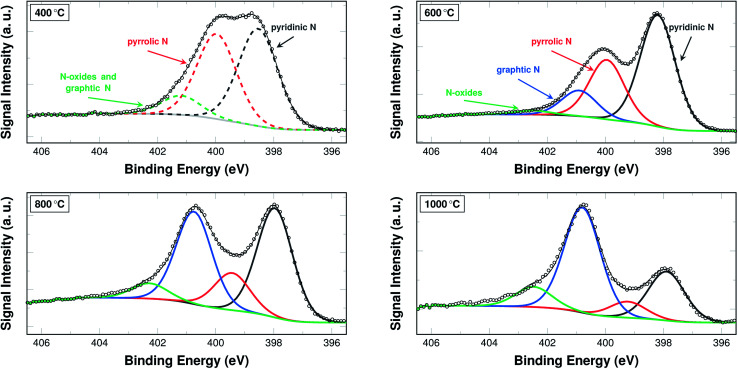
N 1s section of XPS spectra of samples carbonised at 400 °C, 600 °C, 800 °C, and 1000 °C. The spectrum of a sample carbonised at 400 °C was fitted with three peaks, as the signals for N-oxides and graphitic nitrogen could not be deconvoluted in this case (dashed lines). Spectra of samples carbonised at 600 °C, 800 °C, and 1000 °C were fitted using a total of four peaks (solid lines).

The nitrogen content in and of itself is not a sufficient indicator for catalytic activity. Therefore, the bonding situation of the nitrogen on the surface of the fibres is investigated *via* XPS. Fig. 5 shows the development of the signal intensities for binding energies from 395.5 eV to 406.5 eV, which can be attributed to different bonding situations of nitrogen in a (graphitic) carbon structure (additional spectra are shown in Fig. S2 and S3†).

A common approach in attributing the peaks to bonding types is as follows: the peak at 398.7 eV corresponds to pyridinic nitrogen, the peak at 400.3 eV to pyrrolic nitrogen, the peak at 401.2 eV to graphitic nitrogen, and the peak at 402.8 eV to pyridinic N-oxides.^26^ Due to their interaction and the fitting procedure itself, the peaks may shift their position slightly to both higher and lower binding energies.

From a practical point of view, two temperature ranges have to be distinguished. For the samples carbonised below and at 500 °C, the spectra were fitted with only three peaks. In these cases a distinction between the graphitic nitrogen and the N-oxides is not possible, although it may be assumed that the signal stems mostly from N-oxides. The spectra of the samples carbonised at 600 °C and above were accordingly fitted with four peaks, distinguishing between the two species.

**Fig. 6:**
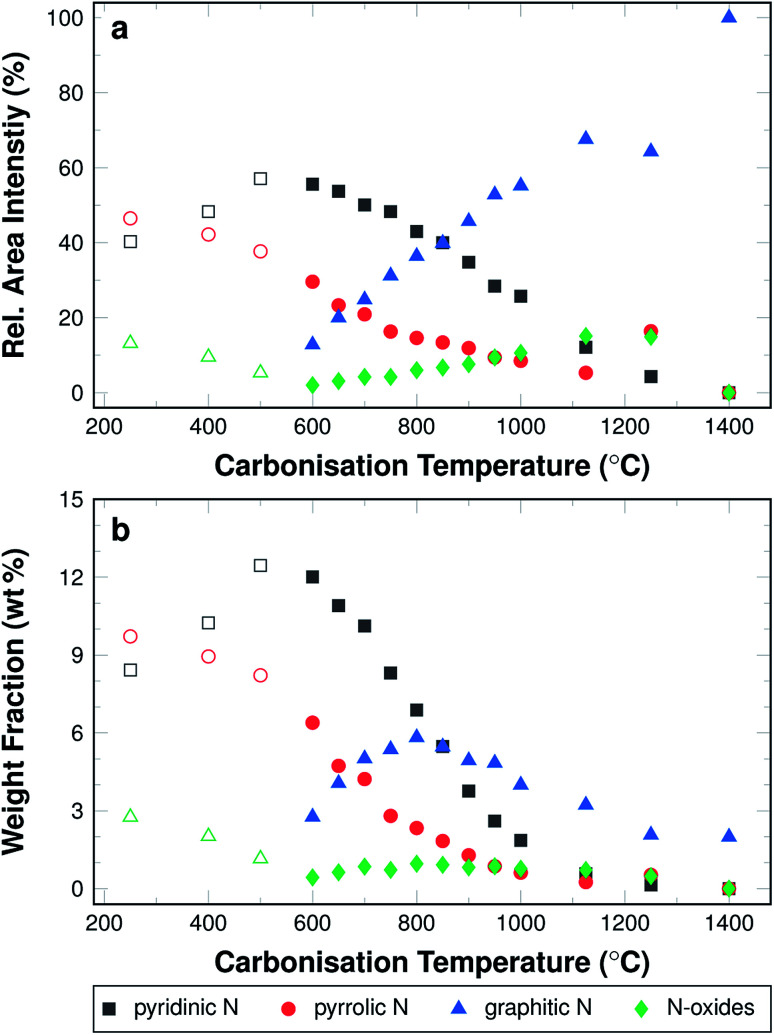
(a) Relative area intensities of XPS peaks attributed to different types of nitrogen. (b) Weight fractions of individual nitrogen species calculated from a combination of XPS and elemental analysis. The open symbols represent the attribution of the peaks for a fitting with three peaks; the closed symbols for a fitting with four peaks.

The amounts of nitrogen species and their development with carbonisation temperature are shown, in detail, in Fig. 6. The development of the relative fraction of each species as results from the area intensities are shown in Fig. 6a. The weight fraction of the nitrogen species determined by multiplying the relative fraction of nitrogen species according to XPS (Fig. 6a) and the nitrogen content from the elemental analysis (Fig. 3) is shown in Fig. 6b.

In the low temperature range up until 600 °C, the signal intensity attributed to pyrrolic and mixed graphitic and N-oxides decrease. In turn, the intensity ratio of pyridinic nitrogen increases from 40.3% to 57.1%. At 600 °C, the signal intensity of pyridinic nitrogen has a maximum, both in terms of relative intensity and weight fraction. In the temperature range above 600 °C, the signal intensity for pyridinic nitrogen decreases from 57.1% at 600 °C to 0% at 1400 °C. The signal intensity of pyrrolic nitrogen decreases throughout the entire investigated temperature range from 46.5% after stabilisation in air at 250 °C to 0% after carbonising at 1400 °C in argon. The signal intensity of graphitic nitrogen, once it can be deconvoluted individually, increases from initially 12.8% at 600 °C to 100% at 1400 °C. The signal intensity attributed to N-oxides that can be identified in this temperature range increases from 2% at 600 °C to 15.1% at 1250 °C, but is then reduced to 0% at 1400 °C.

It is possible that the minimum of the N-oxide signal intensity at 600 °C results from a systematic error of interpretation, when the XPS signal is fitted with three instead of four peaks (Fig. 5). It may in fact be constant in the entire carbonisation temperature range until 1250 °C.

Pyrrolic nitrogen, by definition, occurs in five-fold rings as part of N–H groups, but not in six-membered ones. Pyridinic and graphitic nitrogen is part of six-membered rings, substituting for a carbon atom in the ideal graphite structure. Pyridinic nitrogen occurs along the edges of a graphene layer, graphitic nitrogen will be part of the bulk area. Graphitic nitrogen will disturb the ordering of the material less than pyridinic nitrogen, which causes less disorder than pyrrolic nitrogen. Hence, the XPS results imply an increasing ordering and an ongoing graphitisation along the temperature axis. Pyridinic nitrogen will most likely dominate in structures with many edges. Large amounts of graphitic nitrogen imply larger clusters of graphitic areas, with fewer edges and defects.

The decreasing trend for the weight fractions of pyrrolic and pyridinic nitrogen (Fig. 6b) is more pronounced than for the relative amounts. Graphitic nitrogen shows a clear maximum of 6 wt% in the samples carbonised at 800 °C. At 1400 °C almost all of the nitrogen present in the material is graphitic, albeit this means only 2 wt% of the material. This behaviour of the weight fraction of graphitic nitrogen is explained by the increasing trend of the relative fraction with carbonisation temperature, which is counteracted by the decrease in the overall nitrogen content.

### 3.3 Raman spectroscopy

**Fig. 7:**
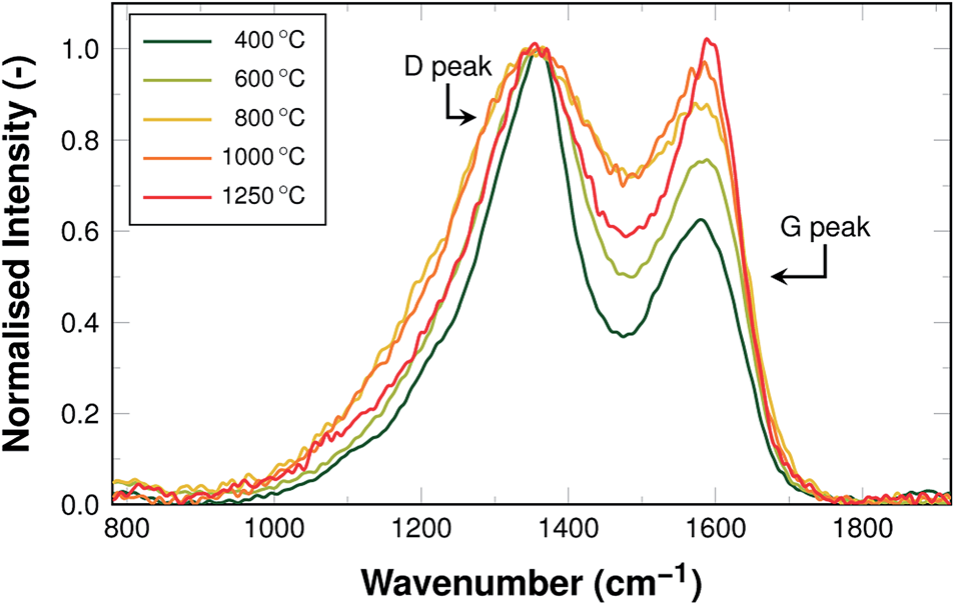
Raman spectra of samples carbonised at 400 °C, 600 °C, 800 °C, 1000 °C, and 1250 °C, normalised to the height of the D peak.

Raman spectra of graphitic carbon materials contain two characteristic peaks. The D peak at around 1355 cm^−1^, the G peak at around 1600 cm^−1^. This is also true for the carbon fibres at hand and the resulting spectra including the D and G peaks are shown in Fig. 7.

In materials composed of carbon only, the Raman signal from 900 cm^−1^ to 1900 cm^−1^ is the result of a superposition of five overlapping bands caused by the different vibrational modes of carbon that exist independently of graphitic ordering.^38,39^ These peaks are broadened in case of nitrogen-doping, as the additional nitrogen leads to additional bands in the Raman shift range from 1630 cm^−1^ to 1670 cm^−1^ as well as defects that enable the A_1g_ mode that is forbidden in ideal graphite.^40,41^ Additionally, oxygen-containing functional groups, which originate from the oxidative stabilisation, cause C=O bands in the range from 1652 cm^−1^ to 1734 cm^−1^.^40^

The resulting total number of bands in nitrogen- and oxygen-doped carbon is very high and the peaks cannot be deconvoluted. The common approach for such cases is a fitting of the two peaks as measured, not taking the several contributions into account individually.^38^ Two variations of this approach are commonly used for fitting: either two Gaussians or a Lorentzian (D peak) and a Breit–Wigner–Fano (BWF) distribution (G peak). The BWF distribution is an asymmetric function, accounting for the additional dopant contributions. The two methods have been discussed at length by Ferrari and Robertson^39^ and in this work, the Lorentz/BWF method was chosen.

**Fig. 8:**
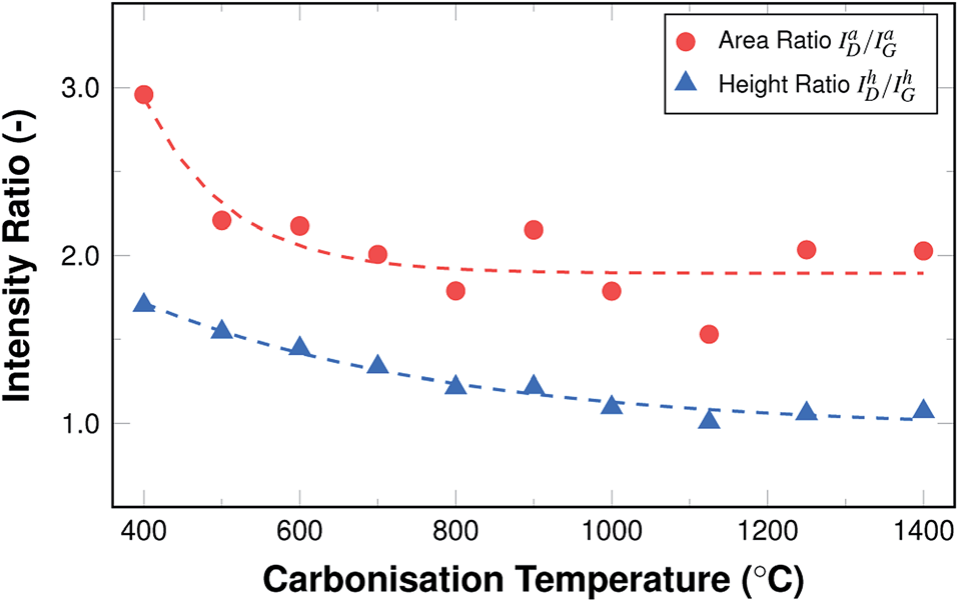
Raman area and height intensity ratios *I*_D_/*I*_G_ as a function of carbonisation temperature.

Fig. 8 shows the resulting development of the ratio of both the maximum height of the fits *I*^h^_D_/*I*^h^_G_ as well as their areas *I*^a^_D_/*I*^a^_G_, both as a function of the carbonisation temperature.

Both ratios display an exponential decay, with the height intensity ratio decreasing from 1.75 to roughly 1.0 and the area intensity ratio decreasing from 2.9 to about 1.9. This reduction of the ratio is related to an increase in the graphitic ordering of the material, fewer defects, and a higher number of unperturbed carbon six-fold rings. The more pronounced decrease of the area intensity ratio can readily be explained by the fact that the overall amount of hetero-atoms decreases with carbonisation temperature, leading to a reduction in peak broadening, especially of the D peak. In other words, the fibres display an increase in structural ordering with increasing temperatures throughout the entire temperature range, although the changes are most pronounced in the lower temperature range up to 600 °C.

### 3.4 Electrical conductivity

**Fig. 9:**
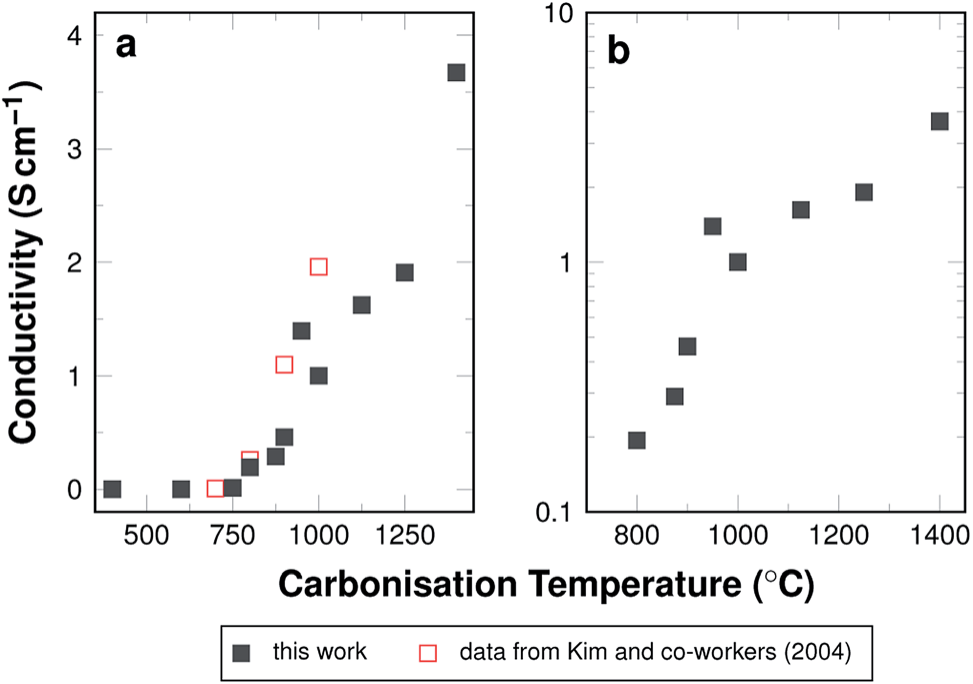
(a) Calculated conductivity as a function of carbonisation temperature. Reference data from Kim and co-workers.^42^ (b) Logarithmic plot of calculated conductivity as a function of carbonisation temperature in a selected range.

In general, electrical conductivity is equivalent to high mobility of charge carriers more specifically electrons. Their mobility may be reduced by *e.g.* grain boundaries, resulting in an increased resistance due to the required charge transfer processes. Electron mobility is enhanced, when electrons are de-localised. In carbon materials this is the case, when a large number of conjugated double-bonds is present, which is especially the case in graphite. The development of the electrical conductivity for the investigated carbon fibre mats is shown in Fig. 9a.

High resistances result in a negligible conductivity in the samples carbonised at low temperatures, *i.e.* below 600 °C. In all likelihood, the π-system has not yet developed in the material and electrons cannot move freely along the fibres. The threshold temperature, above which conductivity is detectable, lies at around 800 °C. This is consistent with the Raman data, which show decreasing *I*_D_/*I*_G_ ratios for samples carbonised at up to 800 °C. This decrease in signal intensity ratio indicates a transformation towards more graphite-like ordering. In terms of increasing conductivity, this makes sense, as the graphite structure is more conductive the fewer structural defects are present. The amount of nitrogen that is not incorporated as graphitic nitrogen decreases, which can be seen in the N 1s XPS data shown in Fig. 6. The structure is, therefore, less perturbed and the conductivity increases.

A logarithmic representation of the conductivity for samples carbonised above 800 °C is shown in Fig. 9b. Between 800 °C and 950 °C the conductivity increases more strongly than between 950 °C and 1400 °C. This effect may be attributed to the incorporated nitrogen, its amount, as well as its bonding situation. Two effects compete in this case. On the one hand nitrogen introduces defects into the ideal graphite system, which is by itself expected to reduce conductivity. On the other hand, nitrogen adds an additional electron to the aromatic system, which is expected to increase the conductivity, as the density of charge carriers in the system is higher.^41^ Also, the defects caused by nitrogen are less influential at higher carbonisation temperatures, where increasing amounts of nitrogen substitute for carbon in the graphitic structure (*cf.*Fig. 6a).

At high temperatures the overall nitrogen content is very low, so that either of the effects vanish and the material behaves like purely carbonaceous fibres. The strong increase of the conductivity is in good agreement with the conductivity of electrospun carbon fibres from PAN reported by Kim and co-workers^42^ as shown in Fig. 9a. Whether the conductivity in this case is governed by the nitrogen doping or the carbon backbone is subject to discussion. For nitrogen-doped carbon nanofibres derived from the decomposition of carbohydrates in ammonia a maximum of conductivity at intermediate amounts of incorporated nitrogen has been reported.^41^ This is different for the electrospun carbon fibres from PAN carbonised in argon, for which the nitrogen content varies with the carbonisation temperature. In our case, the conductivity increases throughout the investigated carbonisation temperature range with the highest values found for samples carbonised at 1400 °C.

**Fig. 10:**
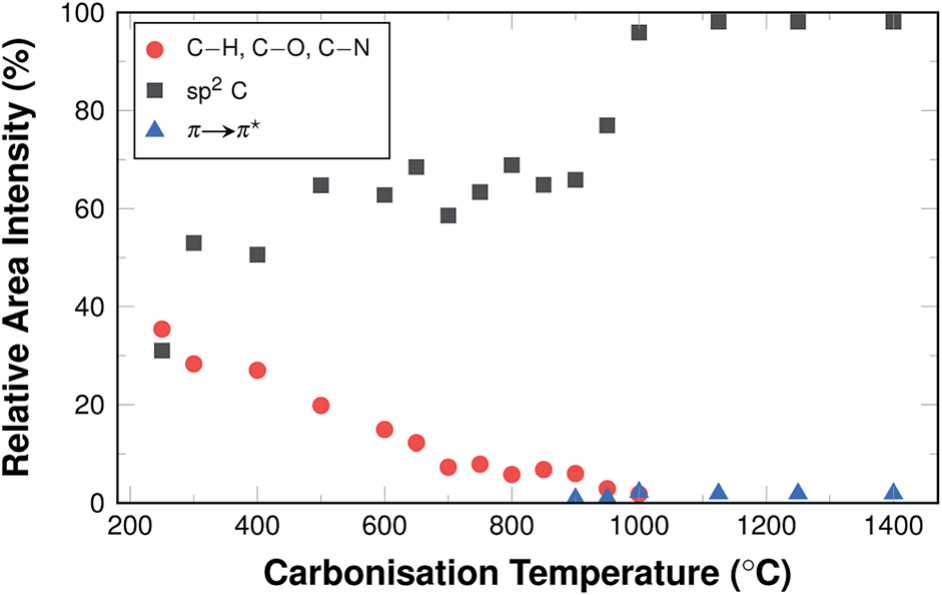
Relative area intensities of XPS peaks attributed to different types of carbon and the π → π^*^ transition. A graph showing all distinguishable species is shown in Fig. S4.†

This structural explanation of the development of the electrical conductivity is further supported by the C 1s range of the XPS spectra (Fig. 10).

The amount of sp^2^-hybridised (*i.e.* graphitic) carbon increases from 30% in stabilised fibres to 60% in fibres carbonised at 600 °C. It remains at this value for carbonisation temperatures up to 950 °C. In samples carbonised at higher temperatures, the amount of sp^2^-hybridised carbon lies at well above 95%. In the same temperature range the π → π^*^ transition becomes detectable and this further supports the theory that the material may display significant conductivity only then.

This behaviour is also consistent with the bonding situation of nitrogen. The XPS results showed that almost all available nitrogen is incorporated as graphitic nitrogen substituting for carbon in the graphitic structure increasingly with carbonisation temperature, which has been discussed above (*cf.*Fig. 6). In general, this means the material is increasingly graphitised, as was already discussed based on the Raman data and the higher the degree of graphitisation, the more pronounced the influence of the conjugated π-system.

Also, it should also be noted that there is an structural overlap of the π → π^*^ transition satellite and the signal of carbonyl and carboxyl groups, which means that in cases where the transition satellite is not accounted for, the other functional groups may be overestimated, while the π → π^*^ transition satellite is underestimated^43^ (C 1s spectra are shown for selected carbonisation temperatures in Fig. S5†).

### 3.5 Electrochemical analysis

**Fig. 11:**
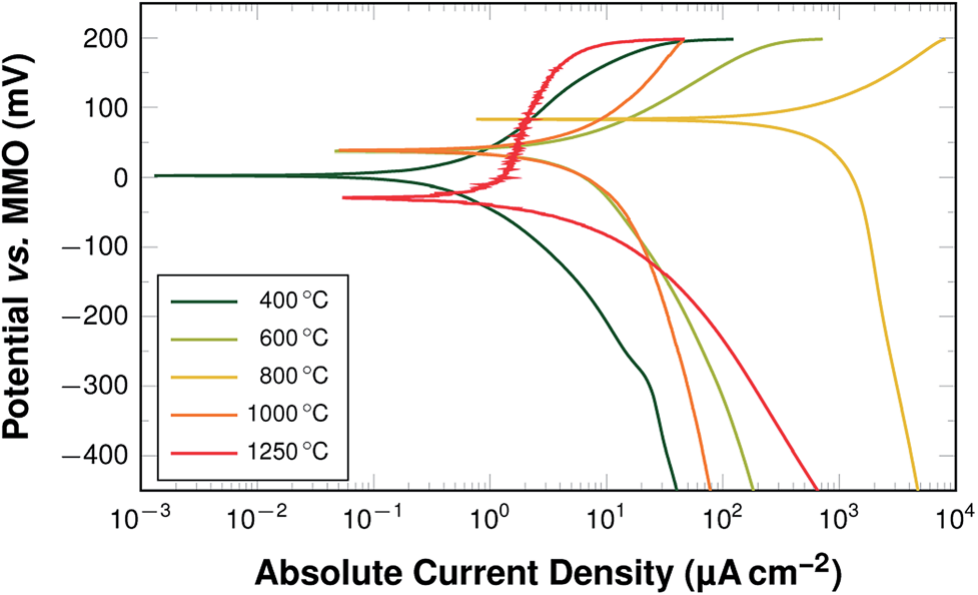
Linear polarisation curves for samples carbonised at 400 °C, 600 °C, 800 °C, 1000 °C, and 1250 °C; after a wetting time of 12 h.

The activity of the PAN-derived fibres was determined using linear potential sweep voltammetry (LSV) in a half-cell setting with 6 M KOH as electrolyte. The characteristic curves obtained from the measurements of samples carbonised at selected temperatures after 12 hours of immersion in KOH are shown in Fig. 11.

A strong dependence of the carbonisation temperature on the electrochemical behaviour is directly visible. This is true for both the open-circuit potential values and the obtained current densities. In order to clarify the trends of the electrochemical behaviour, three key values have been chosen as descriptors:

(1) The open-circuit potential (*E*_OCP_) after 2 h and 12 h of wetting;

(2) The obtained cathodic current at a given overpotential of 20 mV and 100 mV relative to the *E*_OCP_ and;

(3) The required overpotential at cathodic current densities of 1 μA cm^−2^, 10 μA cm^−2^, and 333 μA cm^−2^, corresponding to currents of 3 μA, 30 μA, and 1 mA, respectively.

**Fig. 12:**
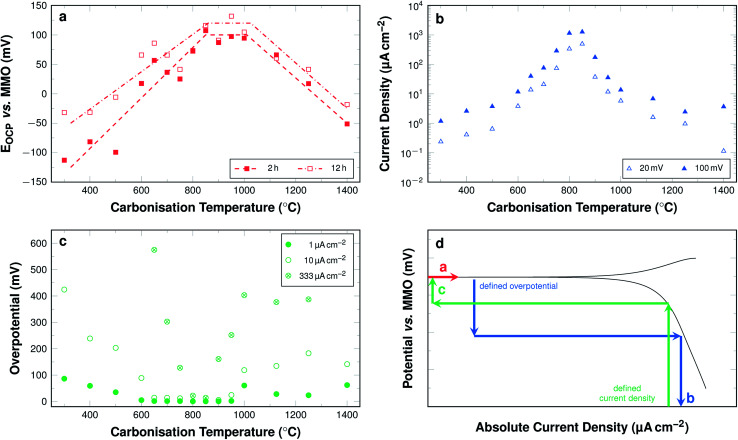
(a) Averaged *E*_OCP_ values after 2 h and 12 h of wetting (dashed lines are guide for the eyes only). (b) Current density at a defined overpotential of 20 mV and 100 mV relative to individual sample *E*_OCP_. (c) Required overpotential to obtain current densities of 1 μA cm^−2^, 10 μA cm^−2^, and 333 μA cm^−2^. (d) Schematic illustration of linear polarisation curve displaying the modes of determination of the three investigated key parameters as shown in Fig. 11a–c.

The development of these key parameters with carbonisation temperature is summarised in Fig. 12a–c and a graphic representation highlighting the mode of value determination is shown in Fig. 12d.

The *E*_OCP_ increases significantly with carbonisation temperatures up to 800 °C (Fig. 12a). The values for fibres carbonised at temperatures between 800 °C and 1000 °C all lie at approximately 100 mV *vs.* MMO. For samples carbonised at even higher temperatures, up to 1400 °C, the potential decreases again to values of about −50 mV *vs.* MMO.

In the lower carbonisation temperature range, the large increase of the potential with the carbonisation temperature may be explained by the fact that at low temperatures the fibres have merely been cross-linked and retain most of the polymer-like properties of PAN. This is supported by the Raman and C 1s XPS data and suggests that substantial carbonisation is only initiated at temperatures above 600 °C. The low potentials for fibres carbonised at low temperatures can also be correlated to the development of the nitrogen bonding situation. Pyrrolic nitrogen and N-oxides are present in large amounts in the fibres carbonised at low temperatures. However, according to the current understanding of oxygen redox chemistry catalysed by nitrogen-doped carbons, they contribute neither to the oxygen reduction nor to its evolution. Graphitic nitrogen, on the other hand, was shown to increase significantly with carbonisation temperature by XPS with a maximum of the absolute amount at 800 °C.

In the temperature range between 700 °C and 1000 °C, the fibres display an increase of only 25 mV. This correlates with the fact that in this temperature range the absolute amount of graphitic nitrogen changes only marginally. In addition to this, the overall content of nitrogen is still relatively high. It decreases from 12.5 wt% to 7.5 wt%, and the fibres display a significant increase in electrical conductivity, as discussed above.

In the high temperature range the potentials decrease. For an explanation, the absolute amount of nitrogen needs to be considered. It drops to almost 0 wt% at 1400 °C. Therefore, the catalytic activity is diminished, as pure carbon does not display substantial inherent catalytic activity towards the ORR.

The general trends are persistent with wetting time. Fibres carbonised at temperatures above 800 °C display almost constant values with wetting time. Fibres carbonised at temperatures between 300 °C and 800 °C display slightly increasing values with wetting time (Fig. 12a). The deviations amount to as much as 75 mV, especially for samples carbonised at low temperatures. The fibres carbonised at temperatures below 500 °C tend to swell, when in contact with the KOH electrolyte, and partially disintegrate during the measurement.

Fig. 12b shows the cathodic current density at an overpotential of 20 mV and 100 mV relative to the respective *E*_OCP_ as a function of the carbonisation temperature. The obtained current densities increase progressively with increasing carbonisation temperatures up to 800 °C. For samples carbonised at 800 °C and 850 °C at an overpotential of 100 mV with respect to the respective *E*_OCP_ the highest current densities are observed. They are about 1.26 mA cm^−2^ for samples carbonised at 850 °C. The values for samples carbonised at temperatures above 850 °C decrease regressively with increasing carbonisation temperatures.

The overall trend is similar for both chosen overpotential values. The current densities are, unsurprisingly, slightly higher with a higher overpotential. This difference of the cathodic current densities at overpotentials of 20 mV and 100 mV for samples carbonised at temperatures below 600 °C is larger than for the other samples. For samples carbonised between 600 °C and 1000 °C the deviation is similar for all temperatures. The obtained current density for samples carbonised at higher temperatures decreases continuously with increasing carbonisation temperatures when only low overpotentials of 20 mV are considered. The values remain constant at around 3 μA cm^−2^ when overpotentials of 100 mV are considered.

The overpotentials in relation to the respective *E*_OCP_ required to obtain cathodic current densities of 1 μA cm^−2^, 10 μA cm^−2^, and 333 μA cm^−2^ are shown in Fig. 12c. For each investigated current density the development of the overpotential can be subdivided into four sections. A decrease, a plateau, an increase, and a second plateau. When focussing on a current density of 10 μA cm^−2^ samples carbonised at 300 °C require an overpotential of 424 mV *vs. E*_OCP_. This value decreases when the samples are carbonised at temperatures up to 650 °C. For samples carbonised in the range between 650 °C and 900 °C the overpotential required to obtain a current density of 10 μA cm^−2^ is close to 0 mV, *i.e.* the current density is readily obtained near *E*_OCP_. The overpotentials are again higher, when samples are carbonised between 900 °C and 1000 °C, where they reach 118 mV *vs. E*_OCP_. The value is similar for all samples carbonised at even higher temperature up to 1400 °C. While the general trends are the same for all investigated current densities, the range of the first plateau, where next to no overpotential is required for significant current densities, is narrower for higher current densities. The lowest required overpotential is found for samples carbonised at around 850 °C even at current densities of 333 μA cm^−2^.

Overpotentials for high current densities display a minimum and likewise the obtainable current densities at given overpotentials display a maximum in samples carbonised at 850 °C. This may directly be related to a maximum in the electrocatalytic activity towards the ORR. However, it cannot be explained by the electrical and morphological properties of the fibres alone. The electrical conductivity increases with carbonisation temperature. The XPS data show that the amount of nitrogen decreases until all nitrogen is removed from the material. None of the properties investigated individually suggest, why the activity displays such a local maximum within the investigated range. Therefore, an explanation can only be found, when the results are related to each other. Summing up the behaviour of the carbon fibres discussed above, five stages can be differentiated by the carbonisation temperature across the employed techniques (Table 1). Overall, these five stages enable a description and explanation of the ORR behaviour throughout the investigated temperature range. The table elucidates especially, how the nitrogen content, but primarily the nitrogen bonding situation influences the electrochemical activity.

**Table 1:** Summary of morphological, structural, electrical and electrochemical properties grouped by carbonisation temperature ranges

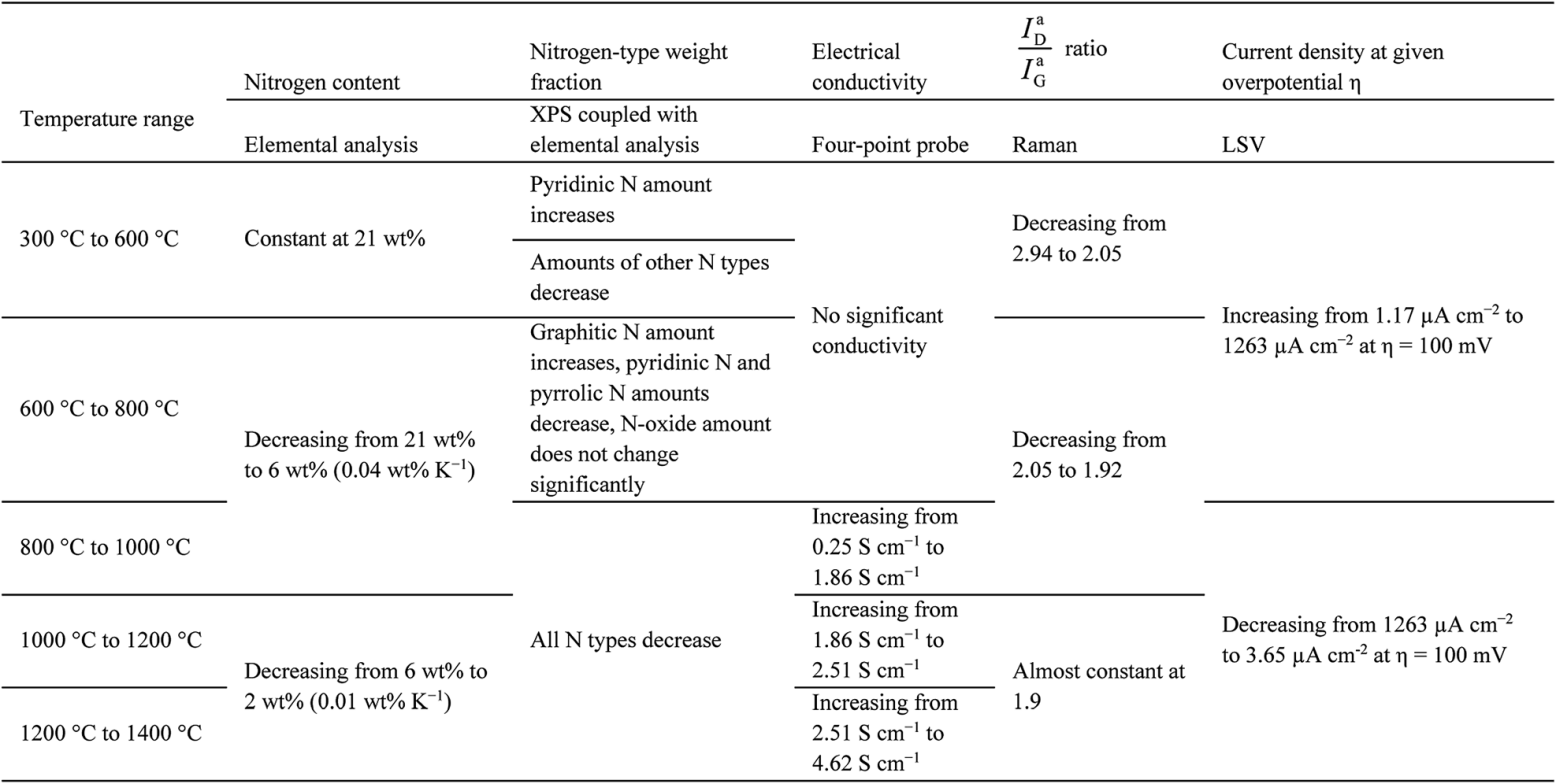

Positive potentials, low overpotentials, and high cathodic currents at given overpotentials correlate with the amount of graphitic nitrogen. It is expected that the potential increases with increaseing amounts of graphitic nitrogen, however, this is not the case. This is likely the result of the reduction of the overall amount of nitrogen, but also due to the fact that increasing graphite-like ordering (as discussed for the Raman results) implies a reduction of edge regions, which are known to be more active.^30^ Furthermore, in this temperature range the amount of pyridinic nitrogen is reduced, which may likewise reduce ORR activity.^22,27^

## 4 Conclusions

The behaviour of electrospun PAN-derived carbon fibres as a framework for air electrodes for aqueous-alkaline metal–air batteries has been investigated in a comprehensive approach. The focus of this investigation was the influence of the carbonisation temperature in a temperature range between 300 °C and 1400 °C on structure, (surface) chemistry of nitrogen, electrical conductivity and electrochemical characteristics of the fibres.

As the carbonisation temperature increases, the amount of nitrogen is reduced from more than 20 wt% to approximately 2 wt%. Likewise, the bonding situation of the nitrogen shifts from pyridinic/pyrrolic to purely graphitic, as was demonstrated by XPS. A correlation between nitrogen content, structure, and electrical conductivity was found. For fibres carbonised at temperatures higher than 600 °C the conductivity of the materials increases. This appears to be the case, once the transformation of the material from polymer-like to graphite-like is initiated. It follows from elemental analysis and Raman spectroscopy, that this transformation is accompanied—if not caused—by the removal of the hetero-species from the structure. The data suggest a dominant influence of the nitrogen removal on this transformation process, compared to a more indirect influence of oxygen and hydrogen.

Evaluation of open circuit potentials, the cathodic currents at given overpotentials, and required overpotentials at given current densities suggest the highest ORR activities for fibres carbonised in the temperature range from 800 °C to 900 °C. The nitrogen bonding situation correlates with the ORR activity of the material, indicating that a high nitrogen content in form of graphitic and pyridinic nitrogen along with sufficient electronic conductivity favours high cathodic current densities. Therefore, our findings support the assumption, that the graphitic nitrogen controls the catalytic activity and that the amount of nitrogen plays a vital role.

Carbon fibres from a PAN precursor show a decent activity towards ORR and feature appreciable electrical properties for applications in aqueous metal–air batteries. The electrochemical behaviour can be influenced by varying the nitrogen content and bonding state *via* temperature adjustment during the carbonisation process.

## Conflicts of interest

There are no conflicts to declare.

## Supplementary Material

RA-009-C9RA03805A-s001
