# Supplementary material for: Carbonisation temperature dependence of electrochemical activity of nitrogen-doped carbon fibres from electrospinning as air-cathodes for aqueous-alkaline metal–air batteries
Source: RSC Adv. 2019 Aug 30;9(47):27231–41. doi: 10.1039/c9ra03805a (PMC9070595; doi:10.1039/c9ra03805a)
Supplement: RA-009-C9RA03805A-s001 [file RA-009-C9RA03805A-s001.pdf]

Electronic Supplementary Material (ESI) for RSC Advances.
This journal is © The Royal Society of Chemistry 2019

Carbonisation Temperature Dependence of Electrochemical Activity of Nitrogen-Doped Carbon Fibres from Electrospinning as Air-Cathode for Aqueous-Alkaline Metal–Air Batteries — Supporting Information

Markus Gehring,,*[ab] Hermann Tempel,[a] Alexandre Merlen,[c] Roland Schierholz,[a] Rüdiger-A. Eichel,[ab] and Hans Kungl[a]

Supplement to DOI: 10.1039/c9ra03805a

1 SEM image of 400 °C sample

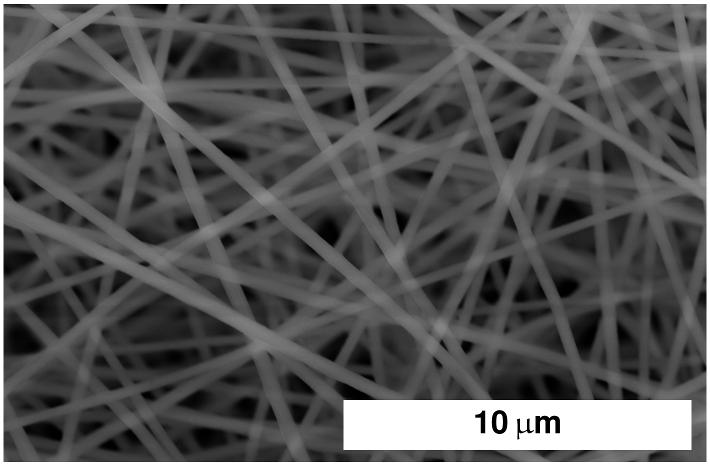

Figure S 1 SEM image of electrospun PAN fibres after stabilisation in air at 250 °C and carbonisation under argon at 400 °C.

2 XPS N1s range

In this section the detailed overview of the N1s range of the XPS spectra is shown in more detail as an addition to Figure 4 of the paper.

Figure S 2 shows the N1s range of the XPS spectra for fibres carbonised at low temperatures. These were fitted with only three peaks, as a differentiation between N-oxides and graphitic nitrogen is not possible for these samples. This is reflected in the corresponding Figures 5 and 6 in the paper.

Figure S 3 shows the N1s range of the XPS for a larger number of samples. The trends summarised in Figure 6 in the paper can be retraced in this more detailed overview.

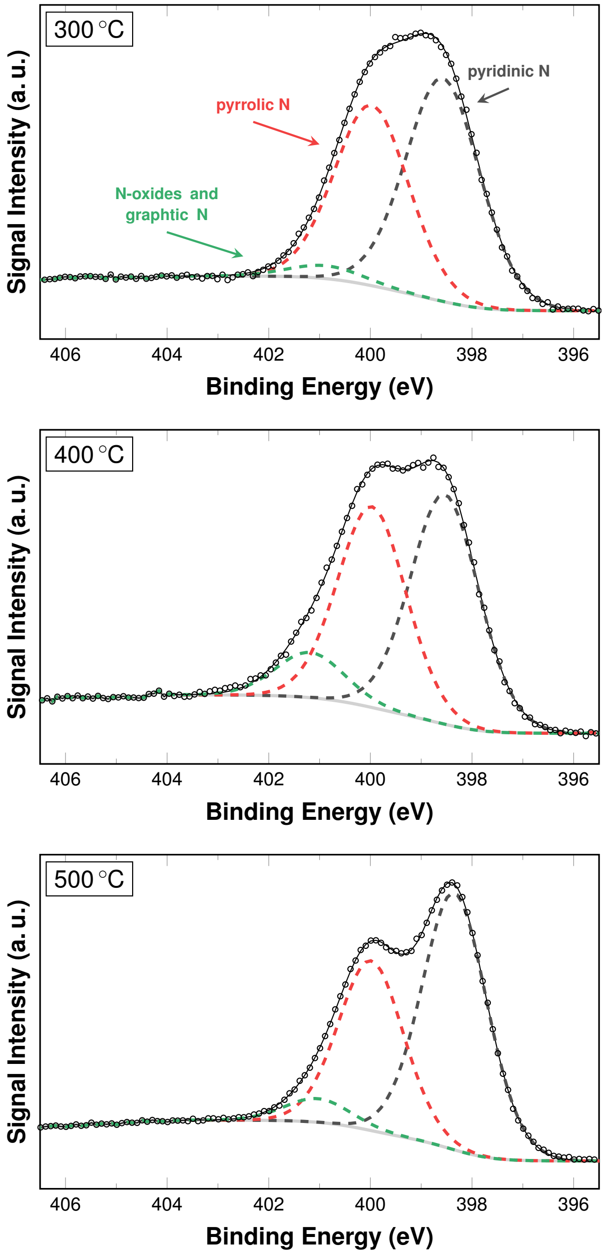

Figure S 2 N1s range of XPS spectra for carbonisation temperatures below 600 °C fitted with three nitrogen species.

[a] Forschungszentrum Jülich GmbH, Institute of Energy and Climate Research – Fundamental Electrochemistry (IEK-9), 52425 Jülich, Germany. Fax: 0049 / 2461 / 61-4155; Tel: 0049 / 2461 / 61-96689; E-mail: m.gehring@fz-juelich.de
[b] Rheinisch-Westfälische Technische Hochschule Aachen, Institute of Physical Chemistry, 52056 Aachen, Germany.
[c] IM2NP, CNRS, Aix-Marseille Université, Université de Toulon, Toulon, France

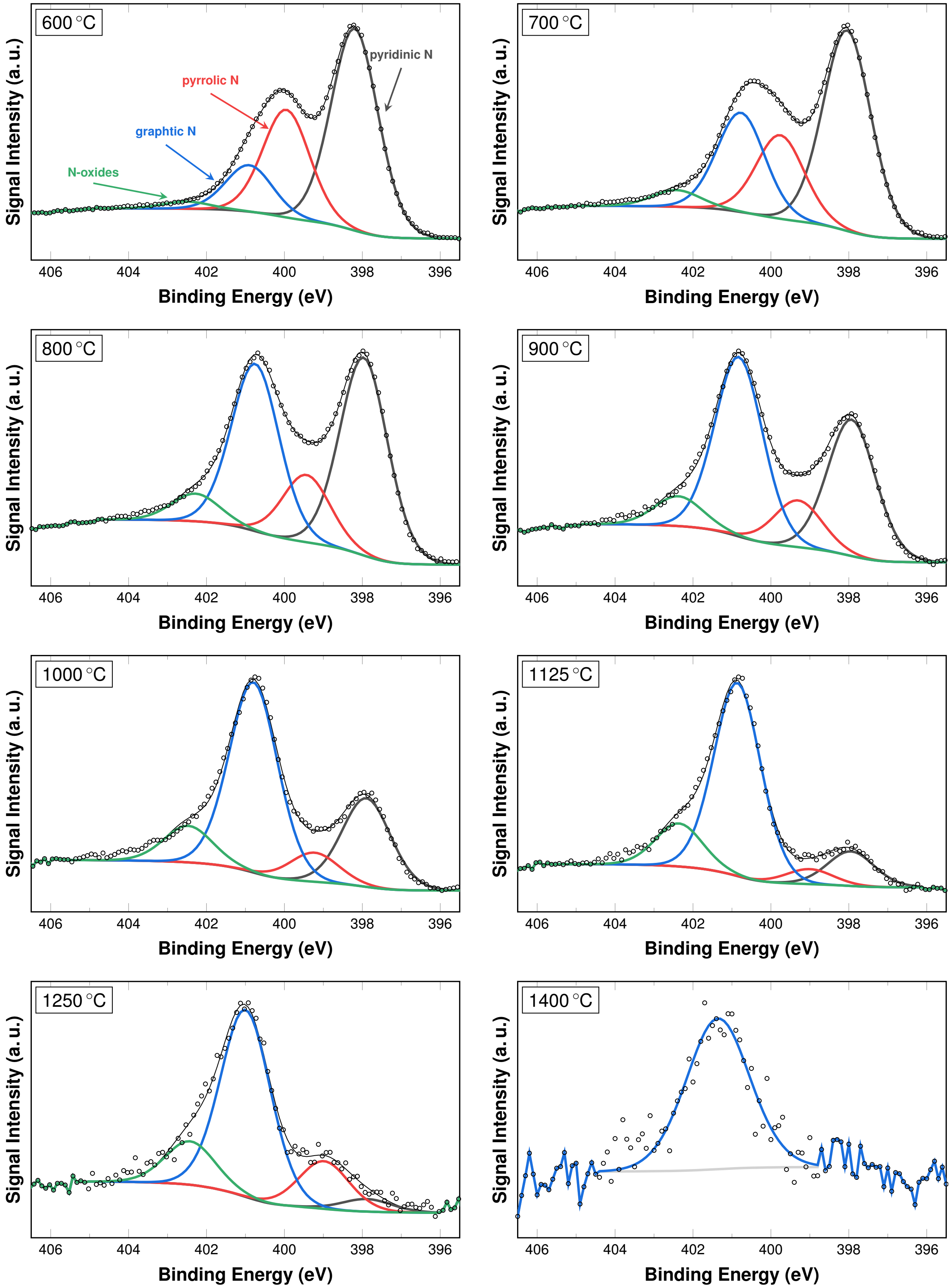

Figure S 3 N1s range of XPS spectra for carbonisation temperatures above 600 °C fitted with four nitrogen species.

3 XPS C1s range

To support the statements made concerning the conductivity and general structure of the carbon backbone, the C1s range of the XPS spectra was analysed. Due to the nature of the C signal, which is a superposition of many C peaks caused by the numerous species that lie in a very narrow range of binding energies, interpreting the C1s data is challenging.

Figure S 4 displays the same overview as Figure 10 with all deconvoluted species.

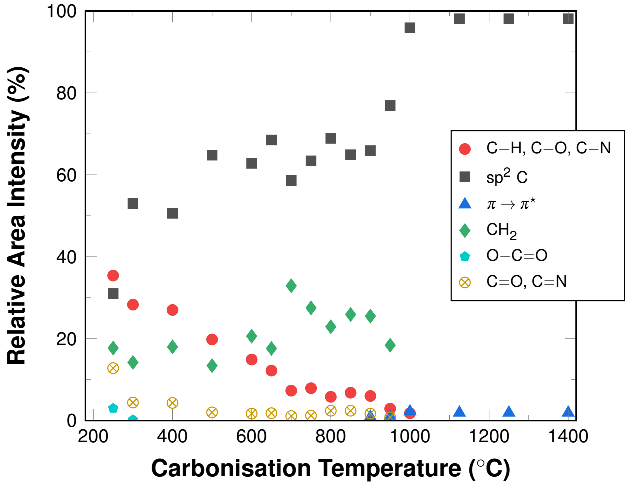

Figure S 4 Relative area intensities of XPS peaks attributed to different types of carbon.

Figure S 5 shows selected spectra that are the basis of Figures 10 and S 4.

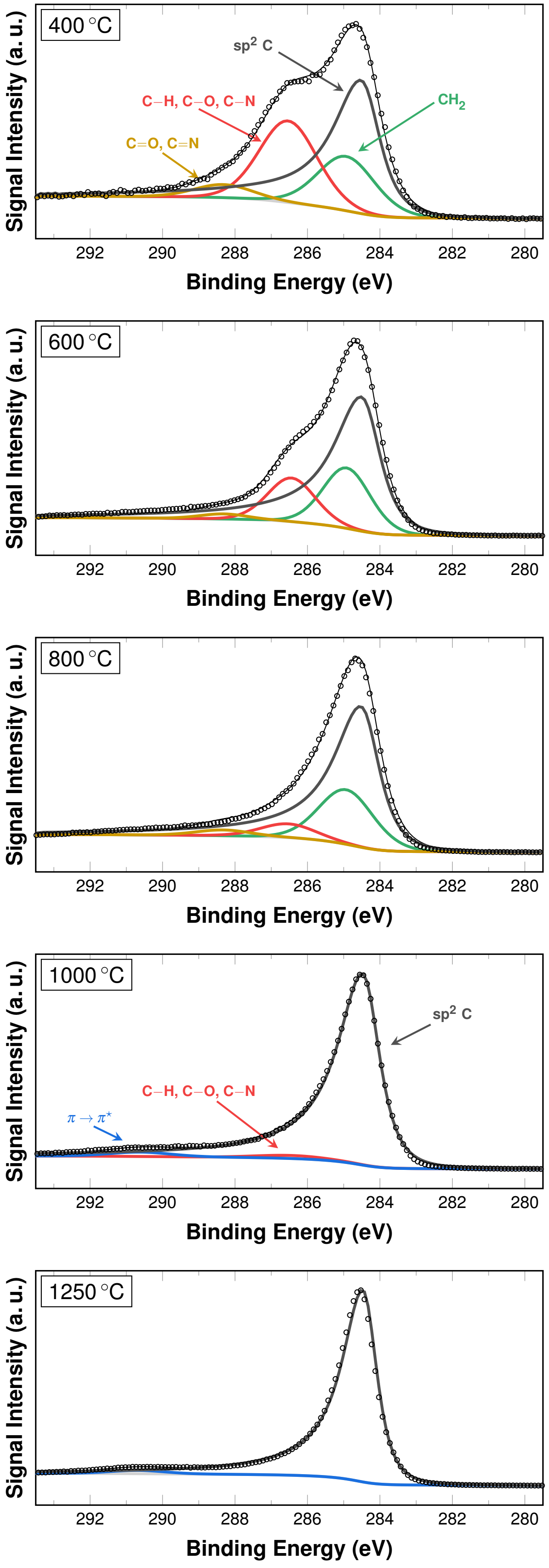

Figure S 5 C1s range of XPS spectra for samples carbonised at 400 °C, 600 °C, 800 °C, 1000 °C, and 1250 °C.
